# Minimally Invasive Mitral Valve Surgery: Long-Term (20-Year) Follow-Up After Right Anterolateral Minithoracotomy

**DOI:** 10.1016/j.cjco.2025.02.001

**Published:** 2025-02-06

**Authors:** Razan Salem, Katharina Fay, Philipp Kaiser, Afsaneh Karimian-Tabrizi, Eva Herrmann, Andreas Winter, Jan Hlavicka, Florian Hecker, Anton Moritz, Thomas Walther, Tomas Holubec

**Affiliations:** a Department of Cardiovascular Surgery, University Hospital and Goethe University Frankfurt, Frankfurt/Main, Germany; b Department of Anesthesiology, Varisano Clinic Frankfurt Höchst, Frankfurt/Main, Germany; c Institute of Biostatistics and Mathematical Modelling, Goethe University Frankfurt, Frankfurt/Main, Germany

## Abstract

**Background:**

Minimally invasive mitral valve (MV) surgery (MIMVS) through right lateral minithoracotomy has evolved as the standard approach for most patients. Data on long-term functional outcomes, however, are rare. We evaluated long-term outcomes after MIMVS through right minithoracotomy for up to 21.6 years.

**Methods:**

From 1997 to 2017, 301 patients with a median age of 57 years (range, 20-81; 54.5% female) underwent MIMVS through right anterolateral minithoracotomy. Follow-up data were evaluated using Kaplan–Meier analyses and competing risk analysis.

**Results:**

A total of 249 patients (82.7%) underwent MV repair, and 52 (17.2%) received valve replacement. Conversion to sternotomy was required in 2 patients (0.8%), and 2 patients (0.8%) suffered perioperative stroke. The 30-day mortality rate was 3.3%. During follow-up, 21 patients required MV reoperation after a mean period of 21.6 ± 0.2 years. The cumulative incidence of reoperation at 5, 10, 15, and 20 years, respectively, was 2.0% ± 0.8%, 4.5% ± 1.2%, 6.0% ± 1.4%, and 7.0% ± 1.6%. The cumulative incidence of recurrent mitral regurgitation ≥ moderate at 5, 10, 15, and 20 years, respectively, was 4.5% ± 1.2%, 11.1% ± 1.9%, 16.4% ± 2.2%, and 20.2% ± 2.7%. The 10- and 20-year survival of all patients was 83.6% ± 2% and 55.0% ± 4%, respectively.

**Conclusions:**

MIMVS can be performed safely with very good perioperative outcomes, a low incidence of mortality, and excellent long-term valve performance.

## Résumé

**Contexte:**

L'accès à la valve mitrale (VM) lors d'une chirurgie mini-invasive de la valve mitrale (CMIVM) par une mini-thoracotomie latérale droite est devenue l'approche standard pour la plupart des patients. Les données sur les résultats fonctionnels à long terme sont toutefois rares. Nous avons évalué les résultats à long terme après une CMIVM par mini-thoracotomie droite sur une période allant jusqu'à 21.6 ans.

**Méthodes:**

De 1997 à 2017, 301 patients d'un âge médian de 57 ans (de 20 à 81 ans; 54,5 % de femmes) ont subi une CMIVM par mini-thoracotomie antérolatérale droite. Les données de suivi ont été évaluées à l'aide d'analyses de Kaplan-Meier et d'une analyse des risques concurrents.

**Résultats:**

Au total, 249 patients (82,7 %) ont subi une réparation de la VM et 52 (17,2 %) ont reçu un remplacement valvulaire. Une conversion en sternotomie a été nécessaire chez 2 patients (0,8 %), et 2 patients (0,8 %) ont subi un accident vasculaire cérébral périopératoire. Le taux de mortalité à 30 jours était de 3,3 %. Au cours du suivi, 21 patients ont dû être réopérés de leur VM après une période moyenne de 21,6 ± 0,2 ans. L'incidence cumulée des réinterventions à 5, 10, 15 et 20 ans était respectivement de 2,0 % ± 0,8 %, 4,5 % ± 1,2 %, 6,0 % ± 1,4 % et 7,0 % ± 1,6 %. L'incidence cumulative d'insuffisance mitrale récurrente ≥ modérée à 5, 10, 15 et 20 ans, respectivement, était de 4,5 % ± 1,2 %, 11,1 % ± 1,9 %, 16,4 % ± 2,2 % et 20,2 % ± 2,7 %. La survie à 10 et 20 ans de tous les patients était respectivement de 83,6 % ± 2 % et 55,0 % ± 4 %.

**Conclusions:**

La CMIVM peut être réalisée en toute sécurité avec de très bons résultats périopératoires, une faible incidence de mortalité et d'excellentes performances valvulaires à long terme.

Since the late 1990s, minimally invasive techniques have been developed to avoid the need for a full sternotomy in mitral valve (MV) surgery.^1^, ^2^, ^3^, ^4^ Although standardized techniques using minithoracotomy, total endoscopic access, and robotic access are gaining widespread popularity, use of the optimal technique during minimally invasive MV surgery (MIMVS) remains imperative.

Over the past 3 decades, until 2017, we performed MIMVS through either upper ministernotomy or right anterolateral mini thoracotomy. After 2017, we adopted minithoracotomy and the total-endoscopic approach as a standard for MIMVS. Only limited data have been collected on long-term outcomes after MIMVS, especially from earlier cases with follow-up data extending beyond 15 years.

We evaluated our single-centre experience with MIMVS through right anterolateral minithoracotomy over a period of 20 years, with particular regard to valve performance (freedom from MV-related reoperation and freedom from recurrent ≥ moderate mitral regurgitation [MR]).

## Materials and Methods

### Study design and population

**Figure 1:**
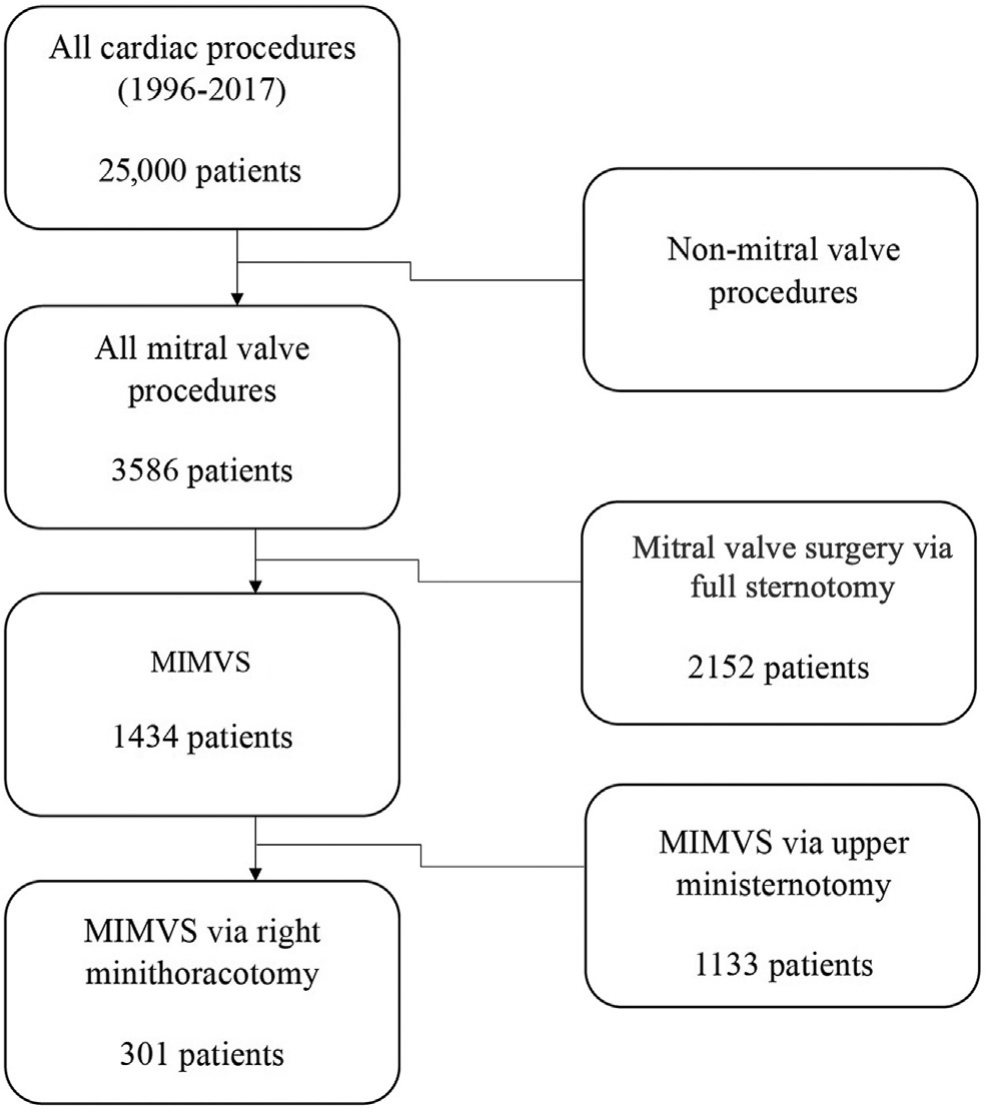
Consort type diagram of patient selection for the study. MIMVS, minimally invasive mitral valve surgery.

From 1997 to 2017, a total of 3586 MV operations were performed in our institution, of which 1434 were minimally invasive—that is, performed through use of an upper ministernotomy or right anterolateral minithoracotomy. All patients who underwent MIMVS through right anterolateral minithoracotomy (n = 301 patients) were included in this study, including those patients who underwent concomitant procedures, such as tricuspid valve repair, closure of a persistent foramen ovale, or a maze procedure (Fig. 1). The method of procedure selection has evolved over the years. Initially, right anterolateral minithoracotomy was considered for all patients amenable to this procedure, except those with known iliac or peripheral artery disease. Later, upper hemisternotomy became the method of choice for low-risk patients (until 2017), owing to the senior surgeon’s preference; and right anterolateral minithoracotomy was selected for moderate-risk patients, and full sternotomy was used for high-risk patients. Due to the novelty of these approaches in the early 2000s, no algorithm for patient selection for a particular procedure was available.

Along with clinical follow-up of the patients who were seen in our outpatient department, we provided valve function evaluation by transthoracic echocardiography. Patients who were not seen personally were contacted by letter or phone. The transthoracic echocardiography results from referring cardiologists were also analyzed (Supplemental Appendix S1).

Exclusion criteria for MIMVS were any of the following: extensive endocarditis; ≥ moderate aortic regurgitation (AR); aortic aneurysm; and poor femoral access. The primary endpoint was defined as MV performance—freedom from MV-related reoperation and freedom from ≥ moderate MR. The secondary endpoint was peri- and early postoperative results and late survival. These endpoints were defined according to the guidelines reported by Akins et al.^5^ Demographic and preoperative data are listed in Table 1.

**Table 1:** Demographic preoperative data

Variable	MIMVS patients (n = 301)
Age, y	57 (46–66)
Sex	
Male	137 (45.5)
Female	164 (54.5)
Atrial fibrillation	108 (60.3)
COPD	23 (8.2)
CHF	51 (18)
Ejection fraction, %	60 (25–85)
NYHA functional class	
I, II	92 (31)
III, IV	205 (69)
Pulmonary hypertension (mPAP > 40 mm Hg)	74 (25.7)
Chronic kidney disease (GFR < 60 mL/min)	44 (15.2)
History of stroke	12 (4.2)
Previous cardiac surgery	20 (6.9)
Etiology	
Degenerative	202 (67.1)
Rheumatic	42 (14)
Endocarditis	18 (6)
Secondary/ischemic	24 (8)
Other	15 (5)

Values are n (%) or median (interquartile range).

CHF, congestive heart failure; COPD, chronic obstructive pulmonary disease; GFR, glomerular filtration rate; MIMVS, minimally invasive mitral valve surgery; mPAP, mean pulmonary artery pressure; NYHA, New York Heart Association.

### Surgical technique

Our surgical technique has been described previously,^6^ although it has evolved in recent years. Initially, we used single-lung ventilation, which has been abandoned in our more recent standard setup.

A small right anterolateral thoracotomy following an approximately 7-cm–long skin incision was performed in the 4th intercostal space (ICS), while simultaneous cannulation of the right femoral vessels for cardiopulmonary bypass was carried out. For the earliest part of this study, femoral cannulation was performed conventionally, through a cut down of the femoral vessels. In the past 5 years, we have used the fully percutaneous cannulation technique with the help of vascular closure devices. In this procedure, the pericardium is opened 3-4 cm anterior and parallel to the phrenic nerve, from the distal ascending aorta to the diaphragm. A straight cardioplegia needle is secured with a purse-string suture at the aortic root. The ascending aorta is cross-clamped using a transthoracic Chitwood Debakey Clamp (Scanlan International, St. Paul, MN) through a 5-mm incision in the 3rd ICS. A Hopkins Optik 30° endoscopic camera (Karl Storz, Tuttlingen, Germany) was placed through a 10-mm port in the 4th ICS.

Antegrade cold blood cardioplegia was administered into the aortic root. The surgical field was constantly flushed with CO_2_ at a 2 L/minute rate through the camera port. The MV was approached through a left atriotomy, and exposure was achieved by placing annuloplasty stitches through both fibrous trigones. After analyzing the valve, repair or replacement was carried out.

The maze procedure was performed with a high-frequency probe (Osypka Medical, Rheinfelden-Herten, Germany) or with a cryoablation probe (CryoICE, AtriCure, Mason, OH) applying the standard lines (pulmonary vein isolation, line to the posterior MV annulus and to the left atrial appendage). Occlusion of the left atrial appendage was achieved with a purse-string and a continuous suture.

### Statistical analysis

All data were collected in a FileMaker (6.0)–based database (Claris, Santa Clara, CA). Data are presented as mean values ± standard deviation, or as median and range for data that are not normally distributed. Normal distribution was evaluated by the Kolmogorov-Smirnov test. Categorical and ordinal variables were expressed by number and percentage of observations. Continuous and discrete variables were compared using a 2-sample *t*-test or the Mann–Whitney *U* test, as appropriate. Overall survival was assessed by Kaplan–Meier curves and compared between groups with a log-rank test. The time to reoperation and dysfunction both were assessed by competing risk approaches using the Nelson–Aalen estimator of cumulative incidence rates, and Gray’s test for group comparisons. A *P*-value < 0.05 was considered to indicate statistical significance. In addition, we analyzed pre-, intra-, and postoperative parameters using univariable and multivariable Cox regression risk analysis to identify predictors for mortality, reoperation, and relevant ≥ moderate MR. A statistical analysis was performed using StatView (Cary, NC), the survival and cmprsk packages in R (version 4.4.2, R Foundation for Statistical Computing, Vienna, Austria), and SPSS (version 25 for MS Windows, IBM, Armonk, NY).

## Results

### Operative data

Intraoperative data are provided in Table 2. Of the 301 patients undergoing MIMVS, 251 patients underwent MV repair, and 52 patients received MV replacement, resulting in a repair rate of 82.7%.

**Table 2:** Surgical procedures and operative times

Variables	MIMVS patients (n = 301)
Mitral valve repair (n = 249)	
Leaflet resection (tri-/quadrangular)	179 (71.9)∗
Chordal replacement†	45 (18.1)
Leaflet plication	4 (1.6)
Commissure plication	9 (3.6)
Sliding plasty	64 (25.7)
Decalcification/patch of the annulus	7 (2.8)
Annuloplasty	
Suture annuloplasty	128 (51.4)
Edwards physio Carpentier ring annuloplasty‡	33 (13.3)
Edwards Cosgrove band annuloplasty‡	64 (25.7)
Classic	19 (7.6)
Medtronic Profile 3D ring annuloplasty§	4 (1.6)
Mitral valve replacement (n = 52)‖	
Mechanical valve	15 (4.9)
Biological valve	37 (12.3)
Concomitant procedure	
Tricuspid annuloplasty	11 (4.4)
Closure of atrial septal defect	14 (5.6)
Maze procedure	
Radiofrequency ablation	35 (14.1)
Cryoablation	6 (2.4)
Conversion to sternotomy	2 (0.6)
Second pump run	13 (5.2)
Operative times, min	
Cross-clamp time	84 (70–101)
Cardio-pulmonary bypass time	146.7 (121–168)

MIMVS, minimally invasive mitral valve surgery.

Values are n (%) or median (interquartile range).

∗ Percentage of repair population.

† Gore-Tex CV-4 (W. L. Gore & Associates Inc, Flagstaff, AZ, USA).

‡ Edwards Physio II and Edwards Cosgrove band (Edwards Life Sciences, Irvine, USA).

§ Medtronic Profile 3D ring ( Medtronic, Mansfield, MA, USA).

‖ Percentage of overall population.

A total of 14 patients required intraoperative revision after repair failure. Reasons for repair failure were a systolic anterior motion phenomenon in 8 cases, and poor ring implantation in 6 patients, which led to valve replacement in 3 of these patients. Of the 52 patients who received MV replacement, 39 (75%) presented with rheumatic valve disease. Every MV repair included annuloplasty, either with a Cosgrove band (25%) or a complete ring (22%). A suture annuloplasty was carried out in 51% of the patients using 2-0 double running suture from trigone to trigone.^7^ Klicken oder tippen Sie hier, um Text einzugeben.

We observed 3 cases of occlusion of the circumflex artery, of which 1 case required coronary artery bypass grafting on the same day as surgery. Reasons for conversion to sternotomy were aortic dissection due to femoral cannulation in 1 case, and ventricular fibrillation and pericardial tamponade in the other case.

### Early postoperative outcomes

The early postoperative data are listed in Table 3. Ten patients died within 30 days of surgery, resulting in a 30-day mortality of 3.3%. Causes of death included low cardiac output (4 cases), sepsis and multi-organ failure (4 cases), and bleeding complications (2 cases). One patient suffered from a permanent neurologic deficit; this was the same patient who had the intraoperative aortic dissection. Another patient suffered from a transitory ischemic attack.

**Table 3:** Postoperative outcomes

Variable	MIMVS patients (n = 301)
30-d mortality	10 (3.3)
Re-exploration for bleeding	23 (7.7)
Myocardial infarction	3 (0.9)
Stroke	2 (0.6)
Postoperative new dialysis	5 (1.7)
Ventilation time, h	12 (10–15)
ICU stay, d	1 (1–1)
Hospital stay, d	7 (7–9)

Values are n (%) or median (interquartile range).

ICU, intensive care unit; MIMVS, minimally invasive mitral valve surgery.

In 4 patients, we observed femoral lymph fistula with superficial groin infection, one of whom required surgical revision. Discharge transthoracic echocardiography revealed a competent valve in 245 of 249 patients after valve repair; 4 patients (1.3%) had mild to moderate residual regurgitation. After MV replacement, 1 patient had trivial paravalvular leak. The mean pressure gradient after MV replacement was 3.75 mm Hg, and 2.7 mm Hg after MV repair.

### Long-term outcomes

Follow-up was complete in 97% of patients (8 patients lost to follow-up), with a median follow-up period of 16.3 years (range, 0.1-23.3 years), making a total of 4229 patient-years.

A total of 21 patients required MV reoperation during the follow-up period. Reasons for reoperation included recurrent MR in 16 cases (of which 2 cases were due to endocarditis), stenosis in 3 cases, and combined pathology in 2 cases. The observed pathologies included recurrent prolapse of the posterior leaflet (8 cases) or anterior leaflet (1 case), retracted posterior leaflet (1 case), perforation due to endocarditis (2 cases), paravalvular leakage (1 case), and degeneration leading to leaflet sclerosis (8 cases).

**Figure 2:**
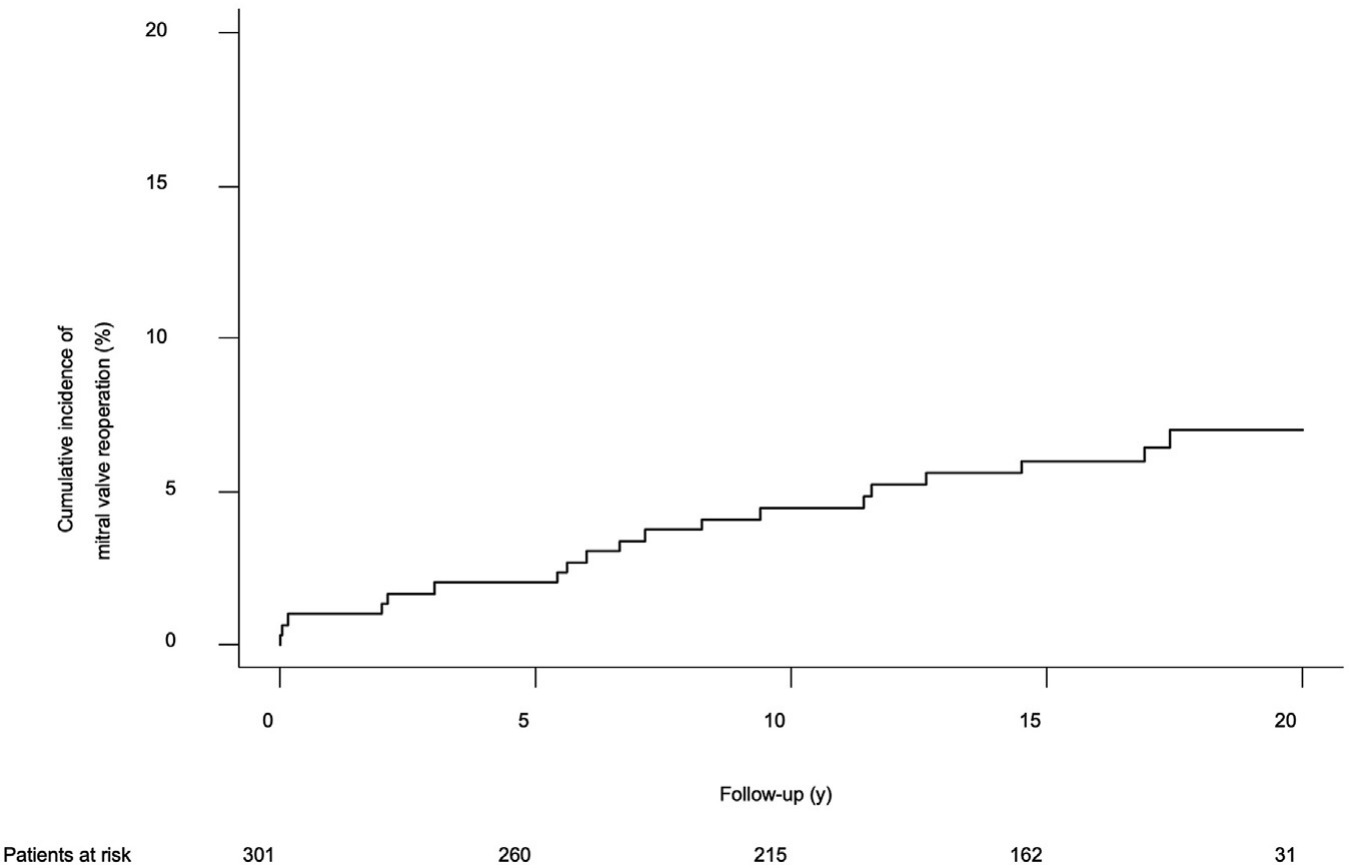
Cumulative incidence of reoperation after minimally invasive mitral valve surgery.

**Figure 3:**
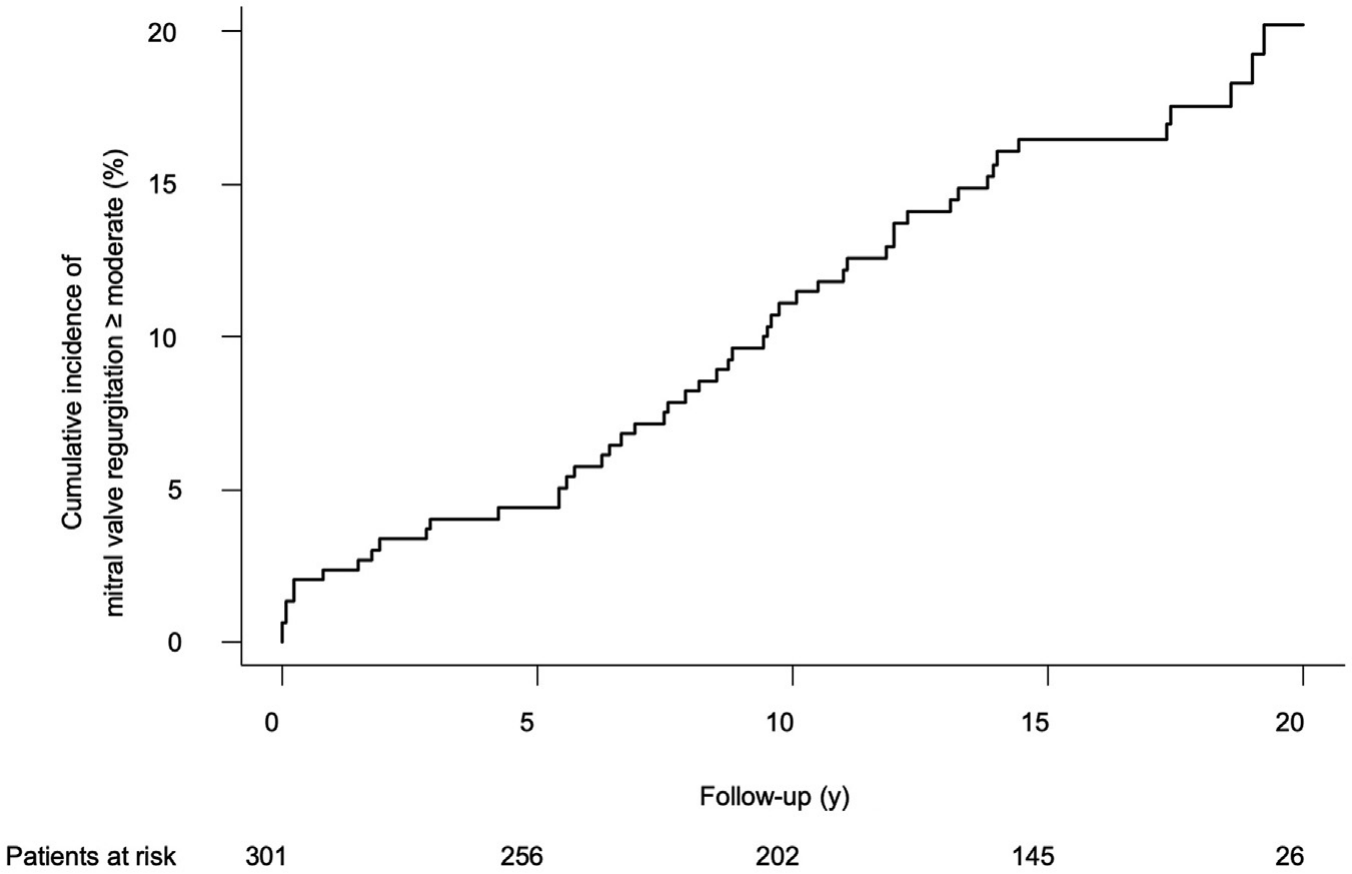
Cumulative incidence of ≥ moderate recurrent mitral valve regurgitation.

In 9 patients, the MV was re-repaired, and the valve was replaced in the 12 remaining redo cases. The cumulative incidence of reoperation at 5, 10, 15, and 20 years, respectively, was 2.0% ± 0.8%, 4.5% ± 1.2%, 6.0% ± 1.4%, and 7.0% ± 1.6% (Fig. 2). No statistically significant difference was found regarding freedom from reoperation (*P* = 0.371) after dividing the overall cohort into repair vs replacement groups (Supplemental Fig. S1). No significant difference occurred in the freedom from reoperation between those patients who had suture annuloplasty vs those who had ring annuloplasty (*P* = 0.316; Supplemental Fig. S2). The overall estimated cumulative incidence of recurrent ≥ moderate MR at 5, 10, 15, and 20 years was, respectively, 4.4% ± 1.2%, 11.1% ± 1.9%, 16.4% ± 2.2%, and 20.2% ± 2.7% (Fig. 3).

**Figure 4:**
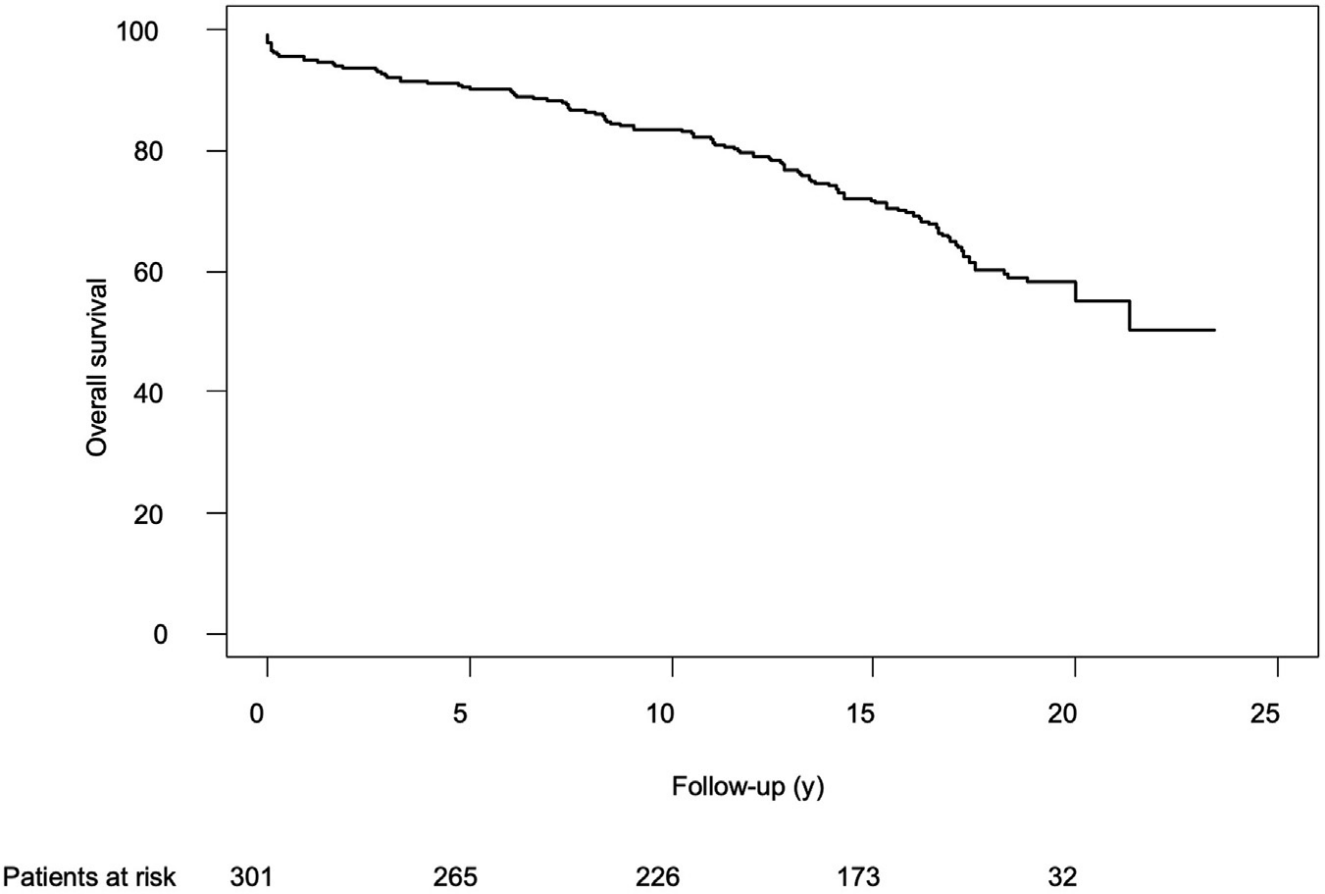
Overall survival after minimally invasive mitral valve surgery.

A total of 109 patients (36.9%) died during the follow-up period, resulting in an overall estimated survival of all patients after MIMVS at 5, 10, 15, and 20 years, respectively, of 90.1% ± 2%, 83.6% ± 2%, 71.7% ± 3%, and 55.0% ± 4% (Fig. 4). No significant difference occurred in survival with MV repair vs MV replacement (Supplemental Fig. S3).

### Risk analysis for survival

Using univariable Cox regression, a total of 16 preoperative, 7 intra-operative, and 7 postoperative risk factors were identified as predictors of mortality (Supplemental Table S1). The multivariable Cox regression analysis identified 3 risk factors—age, previous cardiac operations, and postoperative MR—that were associated with a statistically significant increase in mortality incidence (Supplemental Table S1).

## Discussion

Since the 1990s, much effort has been made to reduce surgical trauma and optimize patient care via the high long-term durability of surgical repair. Excellent long-term results have been published regarding MV repair through conventional sternotomy.^8^, ^9^, ^10^ MIMVS, however, has become the standard approach for MV surgery in many centres. Skepticism has been raised regarding the quality of repair, due to possible “limited exposure” and the technically demanding setting. MIMVS has been shown to be associated with good midterm outcomes.^6^ Herein, we report on long-term outcomes, including valve performance and durability.

One of the major concerns in MIMVS is the presumed lower threshold for MV replacement due to the higher technical complexity of the procedure. Yet we achieved a repair rate of 82.7%, despite including endocarditis (6%), calcified mitral annuli, and rheumatic valvular disease (14%), which are considerably more demanding than degenerative pathologies. Our repair rate is quite comparable to that reported in other publications.^11^ Nissen et al. even reported a higher repair rate in degenerative MV disease in the MIMVS in comparison to that with conventional sternotomy.^12^ Other studies with higher repair rates included only degenerative MV disease.^8^^,^^9^^,^^13^

The quality of our work in MV surgery, specifically in MV repair, translates to a low occurrence of reoperation, as an indicator of valve competence. To analyze the freedom from reoperation as precisely as possible, we chose a competing risk framework, as Kaplan–Meier analysis violates the assumption of independent censoring in the presence of competing events (mortality and reoperation).

We report a low occurrence of MV reoperation—7.0% ± 1.6% at 20 years—which is in line with findings of Glauber et al., who reported a freedom from reoperation of 91.1% at 10 years.^14^ However, due to the use of different analysis methodologies, direct comparison between these results may not be conclusive. McClure et al. reported a series of MIMVSs through lower hemisternotomy and right minithoracotomy of 1000 patients, with a freedom from reoperation of 96% ± 1% at 5 years, 95% ± 1% at 10 years, and 90% ± 3% at 15 years.^15^ Only a limited amount of data has been collected about the long-term results beyond 10-15 years.

Our study encompasses 20 years of experience and a high freedom of reoperation for up to 20 years, which compares to the 20-year freedom of reoperation of 83.3% reported by Feirer et al.^13^ and to the data delivered on conventional MV repair via full sternotomy, as reported by David et al., with a freedom from reoperation of 94% at 20 years.^8^

Surprisingly, we did not find suture annuloplasty to have a high incidence of reoperation or recurrent MR, but we nevertheless ceased to perform this technique, due to its poor reproducibility. In addition, we analyzed the freedom from ≥ moderate MR as a parameter for valve performance. Results were very positive, with a cumulative incidence of only 4.4% ± 1.2% at 5 years, 11.1% ± 1.9% at 10 years, and 20.2% ± 2.7% at 20 years. These results are highly comparable to those in the study by Glauber et al.^14^

The current study shows very good results for 301 MIMVS procedures in an all-comers cohort, with a 30-day mortality rate of 3.3%, which is comparable to that in similar studies. Davierwala et al.,^11^ for example, reported a 30-day mortality for MIMVS of 2.4%. On the other hand, our 30-day mortality rate was slightly higher than that in other studies, such as the 1.1% reported by Glauber et al.,^14^ and the 2% reported by Grossi et al.^16^ We must keep in mind that these data include early experiences with the minimally invasive techniques, performed mostly in the early part of the 20-year period (Supplemental Fig. S5). But most importantly, this procedure was performed mainly in selected higher-risk patients, who were thought to benefit from the minimally invasive access.

Our long-term survival data were also positive and comparable to results in other studies. McClure et al. reported an overall 15-year survival of 795 ± 3% for MIMVS,^15^ which was a study that included a higher percentage of patients with degenerative MV disease (86%, vs our 67% in our cohort). The etiology of MV disease has been suggested to be a mediator of outcomes after MV operations, given that the pathology in degenerative MV disease is relatively localized and simpler to repair, whereas ischemic or rheumatic changes that lead to MV pathologies might be more extensive. However, this possibility could not be demonstrated by a recent examination of the Society of Thoracic Surgery database, in which the etiology of MV disease did not hold independent predictive value for mortality.^17^ Feirer et al.^13^ recently published excellent long-term survival results—91.6% at 10 years and 80.0% at 20 years—in a patient population younger (median age, 55 years) than ours (median age, 57 years). In a Kaplan–Meier analysis (while restricting the data set to patients who survived the first 30 days in a landmark analysis), we found that the survival rate of our patients was comparable to that of a gender- and age-matched general population (Supplemental Fig. S4).

A comparison of the survival rates between patients who underwent MV repair vs those who received MV replacement did not show a significant difference. By contrast, a recent meta-analysis found valve replacement in degenerative MV disease to be associated with a higher mortality rate.^18^ However, our study is not fully representative of degenerative MV disease, as 33% of our cases had nondegenerative etiologies, and most of the replacements were performed in the nondegenerative group. Therefore, we do not conclude from this that repair vs replacement provides the same survival benefit in all MV diseases. Repair remains the gold standard for treating degenerative MV disease, whereas valve replacement might be best in patients with rheumatic disease.

The risk analysis for survival identified age, previous cardiac operation, and residual ≥ moderate MR as predictors for mortality; the first 2 of these factors contribute to a higher European System for Cardiac Operative Risk Evaluation II (EuroSCORE II). Given that younger, asymptomatic patients with few cardiac and noncardiac comorbidities have the greatest long-term survival prognosis, early surgical intervention may be appropriate, even in asymptomatic patients, when the valve seems repairable, according to the current European Society of Cardiology /European Association for Cardio-Thoracic Surgery guidelines.^19^ Moreover, given that residual relevant MR is an independent risk factor for mortality, this factor should be taken into consideration during intraoperative decision-making, in case repair results are not entirely satisfactory.

In our clinical practice, the percentage of MIMVS has increased from 20% in the early 2000s to about 90% today. This 90% rate is higher than the German national average of around 60% for isolated MV operation, as reported on the 2022 German Heart Surgery Report.^20^ MIMVS poses several challenges, such as limited exposure of the surgical field and a smaller access path, that make the process more time-consuming and technically demanding.^21^^,^^22^

### Study limitations

Aside from the obvious limitation of the retrospective nature of this study, our study population is quite heterogenous in regard to the etiology of MV disease. Given that only 67% of the patients presented with primary degenerative MV regurgitation, the repair rate is not entirely representative of the true rate, as the other MV pathologies carry a higher likelihood of the need for replacement beforehand. In addition, the high percentage of suture annuloplasty and the rather low percentage of Neochords (Seramon Chordae Loop, SERAG-WIESSNER, Naila, Germany) used does not reflect the current range of techniques of MV repair. Moreover, patient selection was based partly on surgeon preference and subjective risk assessment.

### Conclusion

MIMVS can be performed with very good long-term outcomes, particularly in regard to MV performance (durability). The procedure offers a low perioperative risk and excellent long-term freedom from reoperation, comparable to that with conventional sternotomy.
