# Supplementary material for: Minimally Invasive Mitral Valve Surgery: Long-Term (20-Year) Follow-Up After Right Anterolateral Minithoracotomy
Source: CJC Open. 2025 Feb 6;7(7):879–86. doi: 10.1016/j.cjco.2025.02.001 (PMC12277840; doi:10.1016/j.cjco.2025.02.001)
Supplement: Supplemental Material [file mmc1.pdf]

SUPPLEMENTAL APPENDIX S1: Patient Management

Prior to surgery, all patients underwent a routine transthoracic echocardiography (TTE), coronary angiography, and/or cardiac computed tomography (CT). Preoperative TTE was primarily obtained by the referring cardiologist (mostly external referrals), and in case of unclear pathology a second preoperative TTE was performed by a senior cardiologist in our hospital specialized in echocardiography, who also performed about 95% of the discharge TTEs.

An intraoperative transesophageal echocardiography (TEE) was performed in all patients to evaluate valve function pre- and post-cardiopulmonary bypass to facilitate the surgical procedure and to assess the immediate result of the MV surgery as well as the left ventricular function during weaning from the cardio-pulmonary bypass and adequacy of de-airing of the cardiac chambers. Postoperatively TTE was performed before discharge from hospital, and at 5, 10, 15 and 20 years after surgery. The echocardiographic classification of residual/recurrent MV regurgitation was as follows: grade 0 – none/trace; grade 1 – mild; grade 2 – moderate; grade 3 – moderately severe; and grade 4 – severe.

The postoperative anticoagulation regimen included initial subcutaneous low-molecular weight heparin for the first days and in parallel oral anticoagulation with vitamin K antagonist. Oral anticoagulation was continued for 6 weeks after an uncomplicated repair and adapted to the needs of atrial fibrillation or implanted prostheses thereafter.

Follow-up was prospectively and systematically collected by means of annually mailed questionnaires or phone interviews and clinical examinations in our outpatient clinic at 5, 10, 15 and 20 years after surgery. For patients not seen personally we retrieved the clinical assessment and echocardiography reports from

the attending (external) cardiologist. The completeness of follow-up was calculated as the portion of the actual observed patient years divided by the maximum of observable patient years as described by Akins et al. [4]. The entry to follow-up was the date of the operation. Follow-up was calculated until: 1. knowledge of death, 2. lost to follow-up, 3. last census date (November 2021). Follow-up was complete in 97% (8 patients lost to follow-up) with a median follow-up of 16.3 years (range 0.1-23.3 years) and accounted a total of 4229 patient-years.

Supplemental Table

Supplemental Table S1. Univariable and multivariable risk analysis: predictors of mortality

	Univariable analysis			Multivariable analysis		
Variables	Hazard ratio	95% CI	p-value	Hazard ratio	95% CI	p-value
Age (per year)	1.084	1.063 – 1.105	0.000	1.096	1.030 – 1.166	0.004
NYHA functional class >II	2.764	1.600 – 4.776	0.000			
Diabetes mellitus	2.571	1.486 – 4.449	0.001			
CHF	2.728	1.791 – 4.156	0.000			
Arterial hypertension	1.803	1.224 – 2.655	0.003			
Pulmonary hypertension	1.954	1.304 – 2.929	0.001			
Coronary artery disease	2.093	1.284 – 3.413	0.003			

Previous cardiac surgery	1.457	1.256 – 1.691	0.000	2.282	1.330 – 3.916	0.003
COPD	2.669	1.535 – 4.641	0.001			
Chronic kidney disease	3.718	2.443 – 5.659	0.000			
Preoperative rhythm	1.896	1.302 – 2.762	0.001			
Urgency of operation	6.538	2.375 – 17.994	0.000			
Cross-clamp time (per min)	1.005	1.001 – 1.009	0.020			
Ventilation time (per hour)	1.002	1.002 – 1.003	0.000			
Concomitant procedure<br>CABG	5.539	1.359 – 22.573	0.017			
Concomitant valve procedure	2.680	1.298 – 5.533	0.008			
Mitral valve pathology	1.243	1.026 – 1,505	0.027			
Inotropes >12h	1.836	1.222 – 2.759	0.003			
Re-exploration for bleeding	2.492	1.417 – 4.380	0.002			
Postoperative MR	1.672	1.144 – 2.445	0.008	2.847	1.397 – 5.803	0.004
Dialysis	15.405	5.995 – 39.583	0.000			
Postoperative pacemaker	5.428	1.324 – 22.251	0.019			

CABG = CABG; CHF = Congestive heart failure; CI = confidence interval; COPD = chronic obstructive pulmonary disease; EF = ejection fraction; LV = Left ventricular; LVEDD = left ventricular end-diastolic diameter; MI = myocardial infarction; NYHA = New York Heart Association

Supplemental Figures

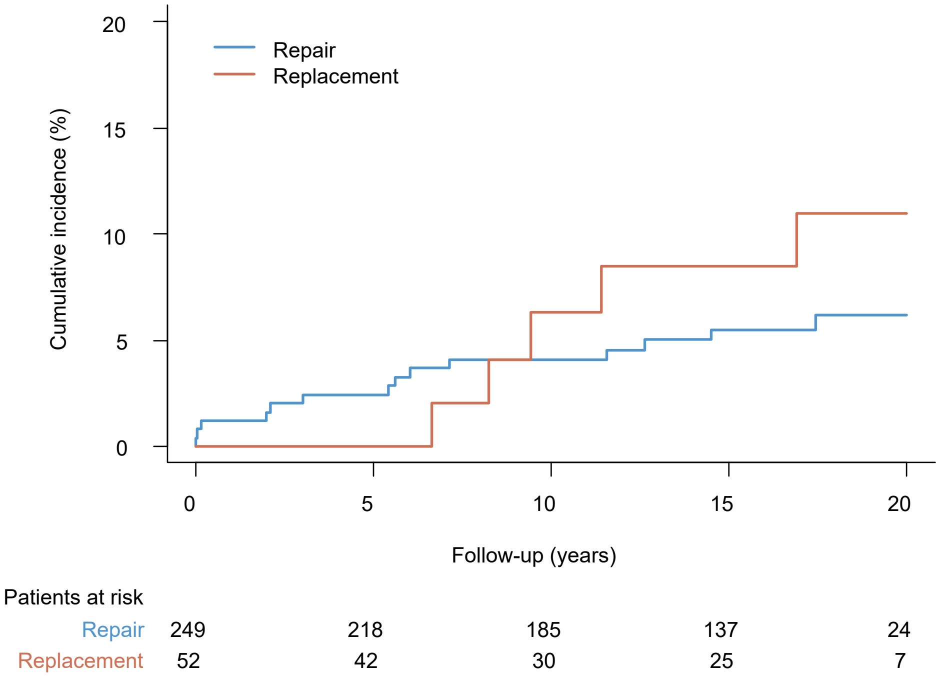

Supplemental Figure S1. Cumulative incidence of reoperation after minimally invasive mitral valve surgery differentiated between patients with mitral valve repair and patients with mitral valve replacement. There was no significant difference.

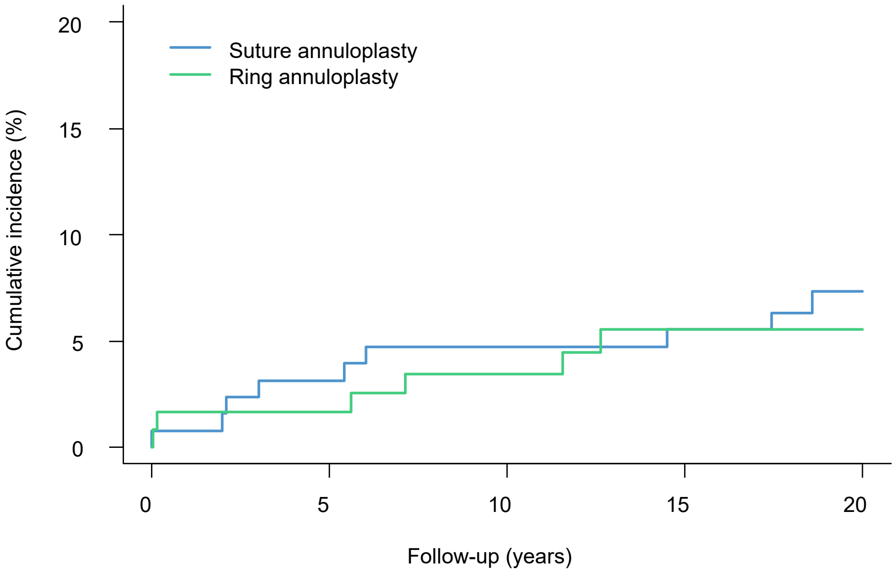

Patients at risk					
Suture annuloplasty	129	114	101	81	23
Ring annuloplasty	120	104	84	56	1

Supplemental Figure S2. Cumulative incidence of reoperation after minimally invasive mitral valve surgery differentiated between patients with mitral valve repair with suture annuloplasty and ring annuloplasty. There was no significant difference.

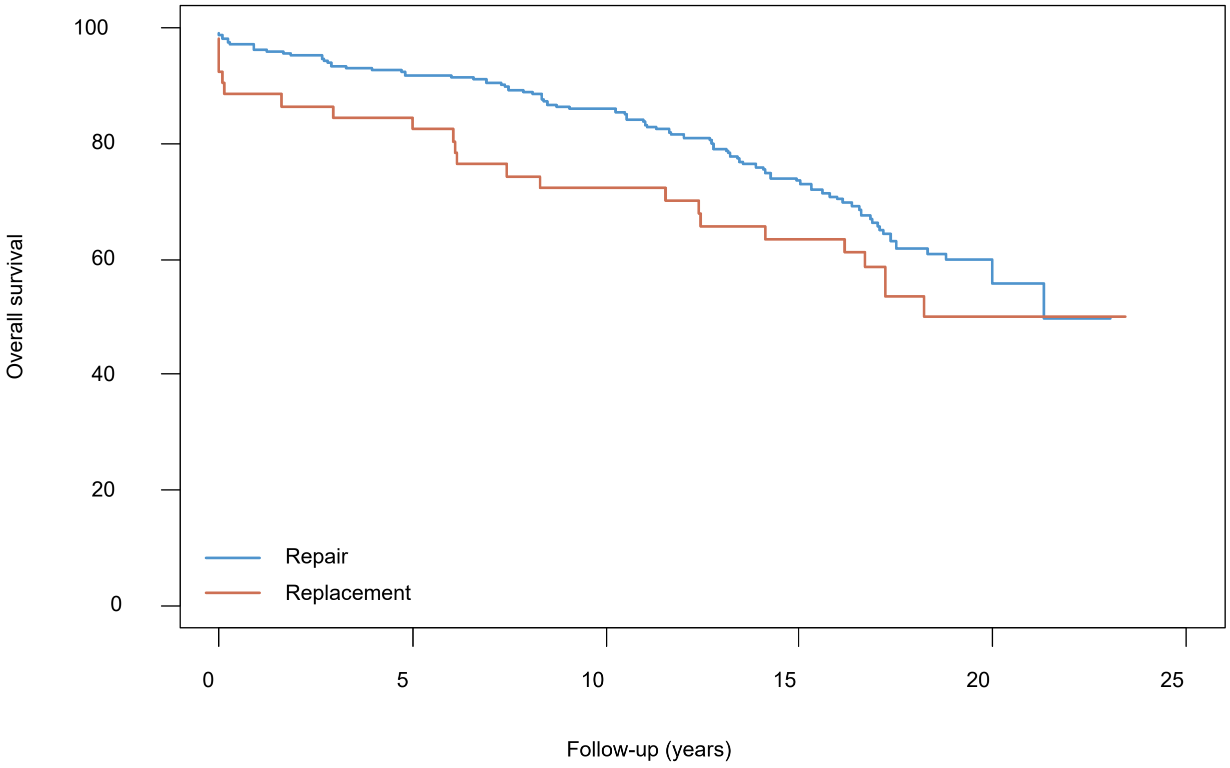

Patients at risk

	0	5	10	15	20
Repair	249	223	193	145	25
Replacement	52	42	33	28	7

Supplemental Figure S3. Overall survival after minimally invasive mitral valve surgery differentiated between patients with mitral valve repair and patients with mitral valve replacement. There was no significant difference.

Landmark Analysis (only patients who survived the first 30 days post-op)

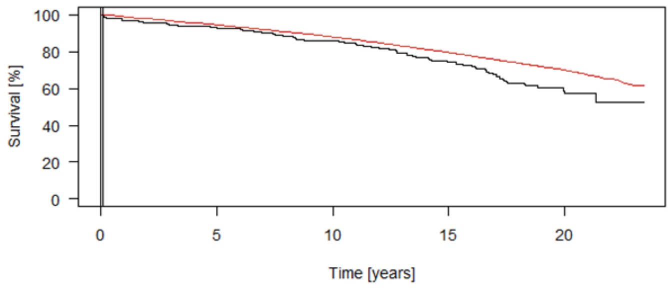

Difference to gender and age matched general population (red line): p=0.1599

	5 years post-op	10 years post-op	15 years post-op	20 years post-op
Survival in study population	93.0% (CI: 90.1%, 96.0%)	85.7% (CI: 81.7%, 89.9%)	74.6% (CI: 69.5%, 80.0%)	57.1% (CI: 50.0%, 65.3%)
Survival in matched general population	94.9%	88.1%	79.6%	70.1%

Supplemental Figure S4. Survival after MIMVS in comparison to gender and age matched population. Post-op: postoperative; CI: confidence interval.

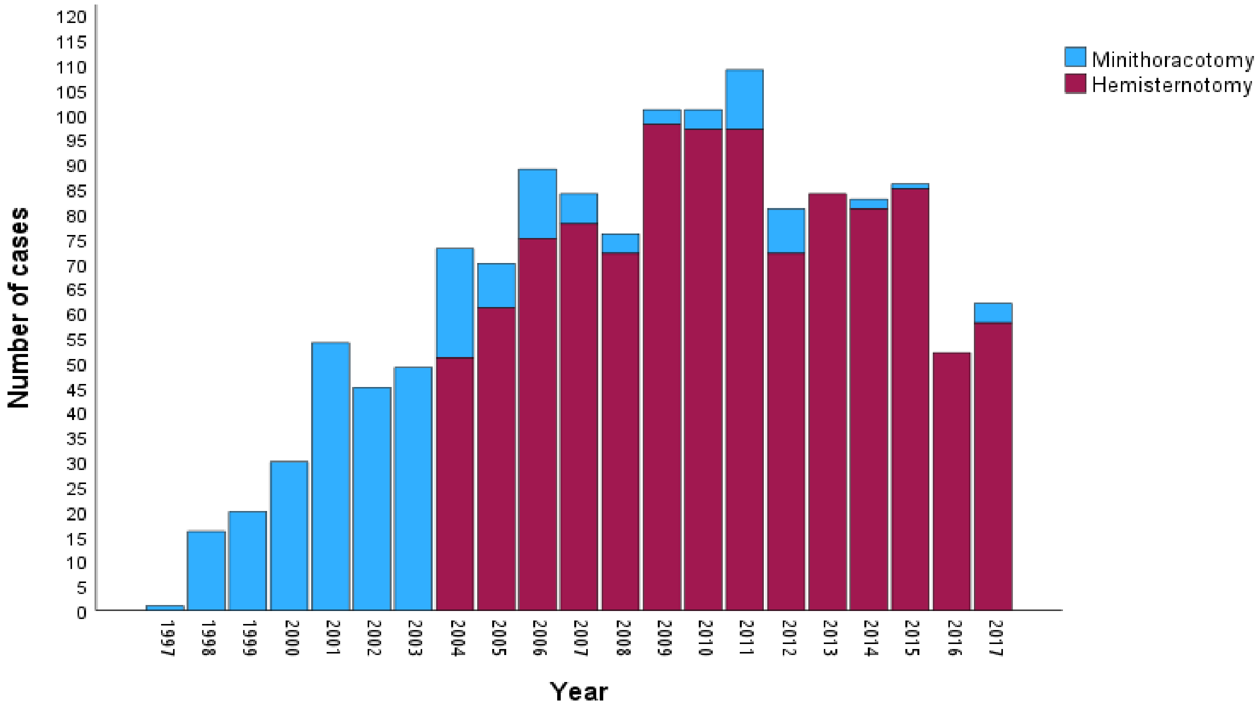

Supplemental Figure S5. Histogram of the use of minithoracotomy versus hemisternotomy for MIMVS over the years; MIMVS=minimally invasive mitral valve surgery.
